# A self-inspected adaptive SMOTE algorithm (SASMOTE) for highly imbalanced data classification in healthcare

**DOI:** 10.1186/s13040-023-00330-4

**Published:** 2023-04-25

**Authors:** Tanapol Kosolwattana, Chenang Liu, Renjie Hu, Shizhong Han, Hua Chen, Ying Lin

**Affiliations:** 1 Department of Industrial Engineering, University of Houston, Houston, USA; 2 School of Industrial Engineering & Management, Oklahoma State University, Stillwater, USA; 3 Department of Information and Logistics Technology, University of Houston, Houston, USA; 4 Department of Psychiatry and Behavioral Sciences, Johns Hopkins School of Medicine, Baltimore, USA; 5 Lieber Institute for Brain Development, Baltimore, USA; 6 Department of Pharmaceutical Health Outcomes and Policy, University of Houston, Houston, USA

**Keywords:** Imbalanced data classification in healthcare, SMOTE-based resampling, Adaptive nearest neighborhood selection, Self-inspection

## Abstract

In many healthcare applications, datasets for classification may be highly imbalanced due to the rare occurrence of target events such as disease onset. The SMOTE (Synthetic Minority Over-sampling Technique) algorithm has been developed as an effective resampling method for imbalanced data classification by oversampling samples from the minority class. However, samples generated by SMOTE may be ambiguous, low-quality and non-separable with the majority class. To enhance the quality of generated samples, we proposed a novel self-inspected adaptive SMOTE (SASMOTE) model that leverages an adaptive nearest neighborhood selection algorithm to identify the “visible” nearest neighbors, which are used to generate samples likely to fall into the minority class. To further enhance the quality of the generated samples, an uncertainty elimination via self-inspection approach is introduced in the proposed SASMOTE model. Its objective is to filter out the generated samples that are highly uncertain and inseparable with the majority class. The effectiveness of the proposed algorithm is compared with existing SMOTE-based algorithms and demonstrated through two real-world case studies in healthcare, including risk gene discovery and fatal congenital heart disease prediction. By generating the higher quality synthetic samples, the proposed algorithm is able to help achieve better prediction performance (in terms of F1 score) on average compared to the other methods, which is promising to enhance the usability of machine learning models on highly imbalanced healthcare data.

## Background

Imbalanced data classification, which refers to the problem of classification when there is an uneven distribution of classes in the training dataset, has been encountered in many healthcare applications, and it is considered as an important topic to discuss in the biomedical data mining area [1–4]. In clinical practice, for example, disease risk prediction models enabled by the classification algorithms are usually built on highly imbalanced datasets with much smaller population of diseased patients compared with healthy individuals. In the area of bioinformatics, classification approach has also been adopted to predict the novel disease-associated risk genes by leveraging the previously implicated disease genes to distinguish patterns between disease-associated and irrelevant gene groups [5]. Due to the limited number of disease genes discovered in biology, the risk gene identification is also an imbalanced data classification problem. However, most classification algorithms were designed based on the assumption of a balanced number of samples for each class. Thus, the classification models built on highly imbalanced datasets are likely to be dominated by the majority class and thus have poor predictive performance on the minority class [6]. It is largely due to a parochial focus on maximizing an average prediction accuracy of the whole training set [7, 8]. A study by [9] on both real and artificial datasets demonstrated that there is an inverse relationship between the performance of a binary classifier and imbalance level of the training data used in model fitting. This behavior has been identified as problematic in imbalanced data classification because the correct classification of the minority class is often of prime interest to the analyst [7]. In other words, the cost of misclassifying the occurrence of a minority sample is substantially greater than the cost of misclassifying the majority class [7].

The performance of classification models built on highly imbalanced datasets, can be improved either at the algorithmic or data pre-processing level [10]. At the algorithmic level, an improvement involves using techniques such as cost-sensitive learning to combat the prediction bias on minority class [7]. Cost-sensitive algorithms, for example C4.5 [11], provide improved classification performance by assigning higher misclassification costs to the minority class. However, the implementation of cost-sensitive algorithms is difficult if there is no domain/prior knowledge on the misclassification costs of the minority and majority classes. Improvement on the data pre-processing level, on the other hand, involves the use of resampling techniques to balance the ratio between minority and majority class samples in the training dataset prior to model learning [12]. The resampling techniques are popular in solving imbalanced data classification problem as they require less prior knowledge and are flexible to be used together with various classification algorithms [13].

Two commonly used resampling methods are downsampling and oversampling [12]. The downsampling works by randomly selecting a subset of the majority class as training dataset so that the balanced ratio between two classes is obtained. Because of the randomness in selecting training data and the sacrifice of training sample size, the downsampling algorithms usually lead to poor and unstable performance. The oversampling, on the other hand, boosts the size of minority class by replicating the minority samples with a random noise. Since there is a replication process which increases the number of training data, it requires more computational cost [7, 14]. Also, as the replicated dataset is too similar to the original data, it does not provide new information and might result in the overfitting issue [14, 15].

To mitigate the limitations in downsampling and oversampling, the Synthetic Minority Over-sampling Technique (SMOTE) algorithm was proposed [16]. It works by selecting samples using *k*-nearest neighbors that are close in vicinity, drawing a line between the selected samples and generating a new sample at a random point along the line. Although the SMOTE algorithm has the ability to generate more minority data for enhancing the classification accuracy, there are some limitations with the quality of the generated samples. First, SMOTE generates data based on the nearest neighbors acquired from the *k*-nearest neighbor algorithm, but it is usually hard to identify the real valuable neighbors when the classes of data are not well separated [16, 17]. Second, to force the decision boundary of the minority class to become more general, it oversamples the minority class by randomly generating samples between each minority data and its nearest neighbors [18]. Unfortunately, SMOTE often generates “uncertain” minority samples, which are on the edge of the class or even belong to the wrong class. The “uncertain” minorities lower the confidence and overall performance of the classification algorithms.

To improve the quality of generated samples from SMOTE and enhance its performance on real dataset, several extensions have been developed. A Borderline-SMOTE or BSMOTE was built to remove the noisy minority samples that have all the neighbors from the majority class [16]. Another extension of SMOTE is the FRIPS-SMOTE algorithm, which was built to obtain high quality samples by utilizing Fuzzy Rough Imbalanced Prototype Selection (FRIPS) technique which cleans data based on the noise threshold [19]. A SMOTE-TL algorithm was also proposed to clean the noisy majority samples which are connected with the minority class by Tomek links [20]. However, it may not be effective when there are noisy minority samples fall into the majority class [21]. Also, similar to SMOTE, it does not remove the low-quality synthetic samples that are not distinguishable from the majority class. The Safe-level-SMOTE was expanded from SMOTE by assigning a safe level to each sample based on the number of minority samples around it [16]. Hence, the resampled data is only generated in the safe regions. The Density Based SMOTE or DBSMOTE is another extension of the BSMOTE algorithm to improve the classification accuracy for both majority and minority classes [16]. The resampled data is generated based on a shortest path from each minority sample and the center of the minority class. However, the existing SMOTE algorithms either ignore the quality of nearest neighbors selected to be resampled or lack of an evaluation metric to quantitative measure the quality of resampled data, and hence are not able to maximally improve the classification accuracy.

In this paper, we proposed a novel self-inspected adaptive SMOTE (SASMOTE) model that utilizes the adaptive nearest neighborhood selection algorithm to identify “visible” nearest neighbors which tend to generate solid minority class samples. Also, an uncertainty via inspection approach is introduced in the SASMOTE model to filter out the low-quality synthetic data that are not distinguishable with the majority class. The structure of this paper is organized as follows. The proposed self-inspected adaptive SMOTE model is described in the method section. Result section presents two real-world case studies of applying the proposed method to risk gene discovery and fatal congenital heart disease (CHD) prediction. Finally, the conclusion of this paper and discussion of future work are provided in the discussion section.

## Methods

**Fig. 1:**
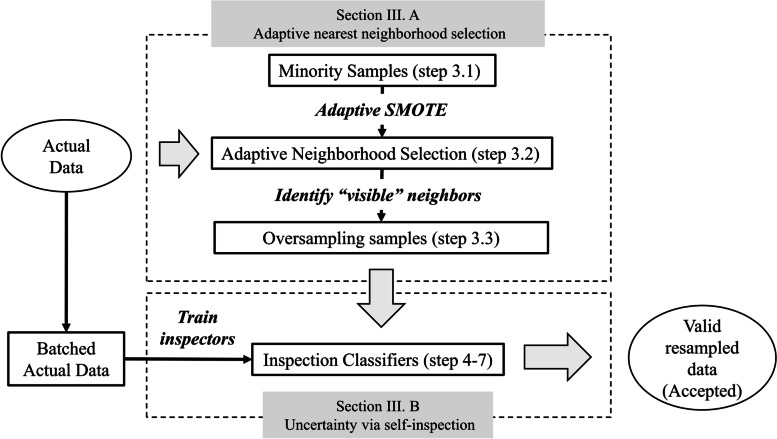
The overall framework of the proposed self-inspected adaptive SMOTE (SASMOTE). The annotation numbers in the brackets correspond to the algorithmic steps shown in Fig. 3

The overall framework of the proposed method is illustrated in Fig. 1, which includes two steps. First, the proposed SASMOTE method generates the minority data based on an adaptive nearest neighborhood selection algorithm, which enforces the usage of “visible” neighbors to generate minority samples. Second, an uncertainty via self-inspection approach determines the quality of resampled data and rules out the “uncertain” minority samples.

### Adaptive nearest neighborhood selection

The conventional SMOTE-based resampling algorithm consists of two steps: the first step searches the *k*-nearest neighbors (KNNs) for each minority data and the second step generates resampled data randomly from the lines between minority data and its KNNs. One of the most significant limitations is that the quality of the resampled data can be low when the minority data are too far away from their KNNs or the neighbors fall into the other classes. To enhance the quality of resampled data, the proposed SASMOTE method conducts an adaptive neighborhood selection algorithm to identify the “visible” neighbors for resampling, which can generate higher quality resampled data than the conventional KNNs approach used in SMOTE algorithm. The idea of “visible” neighbors is inspired from [22] where the neighborhood selection algorithm is applied to avoid the long edge connections between neighbors. Formally, denote the KNNs of a minority sample *x* as $\text {KNN}(x)$, a sample *y* is regarded as a “visible” neighbor of *x* if there is no other neighborhood in $\text {KNN}(x)$ that separates *y* and *x*. In the other words, the angle between edges *xz* and *yz* for every neighbor *z* in $\text {KNN}(x)$ is always acute. The set of “visible” neighbors of *x*, denoted as $\text {VN}(x)$, is a subset of its KNNs with the following formal definition:

**Definition of visible neighbors:** A sample *y* is said to be a visible neighbor of *x* if there is no other neighbor *z* that can separate *y* and *x*, which is expressed as $\langle x - z,y - z \rangle \ge 0, \forall z \in \text {KNN}(x)$. The set of visible neighbors of *x* is defined as

1 $$\begin{aligned} \text {VN}(x) = \{y \in \text {KNN}(x) \mid \langle x - z,y - z \rangle \ge 0, \forall z \in \text {KNN}(x)\} \end{aligned}$$

**Fig. 2:**
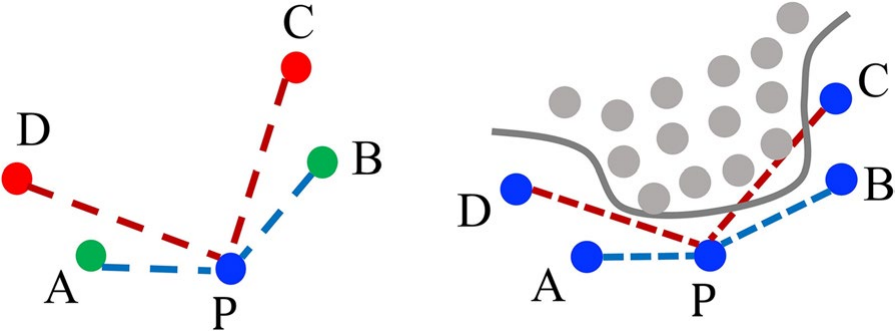
**A** Illustrations of visible neighbors (green dots A and B) and invisible neighbors (red dot C and D). **B** Effects of samples generated from visible neighbors (blue dash lines) and invisible neighbors (red dash lines). Data points randomly generated between P and its invisible neighbors are likely to fall into the other class, which can mislead the classification model toward a biased decision boundary

An exemplary dataset with five samples is illustrated in Fig. 2 (a), where the KNNs of a minority sample *P* is the set *A*, *B*, *C*, *D*. Among the KNNs, the points *C* and *D* are considered as invisible because the angle between the edges *AP* and *AD* and the angle between *BP* and *BC* are obtuse. *A* and *B* are two visible neighbors of *P* identified from the above definition. Then, the proposed method generates resampled data randomly from the lines between minority data and its “visible” neighbors, denoted by the blue dash lines in Fig. 2 (a). To avoid these long edge connections with invisible neighbors, the resampled data are more likely to fall within the minority class when the class is not convex. Specifically, a binary classification problem with nonlinear decision boundary, denoted by the gray curve, is shown in Fig. 2 (b). The minority samples, denoted as the blue dots, lie outside the decision boundary and form a nonconvex set. Based on the definition, *A* and *B* are visible neighbors of sample *P*, *C* and *D* are invisible neighbors. Data points randomly generated between *P* and its invisible neighbors (denoted by red dash lines) are likely to fall into the other class, which can mislead the classification model toward a biased decision boundary. On the other hands, the data generated between *P* and its “visible” neighbors neighbors (denoted by the blue dash lines) are more likely to fall within the minority class. Hence, the resampled data should be generated from the lines between *P* and its “visible” neighbors only.

### Uncertainty via self-inspection

Quantifying the quality of training data is critical for classification model performance. Different statistical metrics have been developed to measure the data quality in classification through its uncertainty, such as the direct uncertainty prediction, Bayesian techniques, and uncertainty via classification [23, 24]. The uncertainty via classification approach assigns a scalar uncertainty score to each sample representing the amount of expert disagreement on the label of this sample. In the resampling schema, we propose an uncertainty via inspection approach which considers the uncertainty as how likely the resampled data fall into the majority or wrong class. The higher probability indicating a higher uncertainty of the resampled data. To estimate the uncertainty score of each resampled data, a set of inspectors or classifiers are trained to predict its labels. The uncertainty score then is estimated as the number of inspectors that classify the resampled data to the majority or wrong class.

**Fig. 3:**
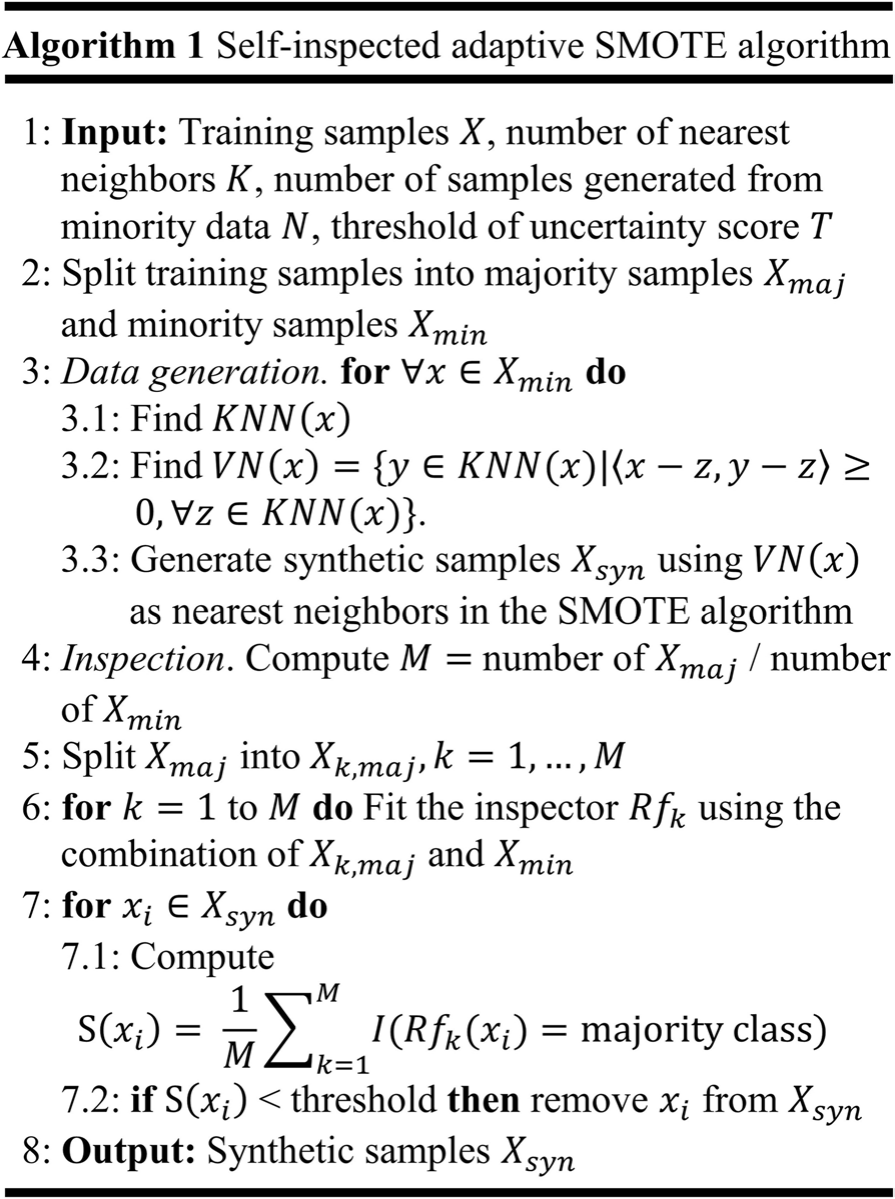
The SASMOTE algorithm

Formally, given a resampled data generated from the adaptive nearest neighbor selection algorithm ($x_i$) and *M* inspectors $Rf_1,\ldots ,Rf_M$, Eq. (2) which represents the uncertainty score of this sample is defined as:

2 $$\begin{aligned} S(x_i) = \frac{1}{M} \sum \limits _{j=1}^{M} I(Rf_j(x_i) = \text {majority class}) \end{aligned}$$

where $I(\cdot )$ is an indicator function. The resampled data with uncertainty score higher than a predefined threshold is regarded as low-quality data and filtered out in the proposed SASMOTE algorithm. The inspectors are trained from M batched training samples. Specifically, the batched training samples are obtained by dividing the majority class to M subsets and combining each subset with the minority class. The number of inspectors controls the size of majority class in each batched training data. To achieve balanced data in each batch, the number of inspectors (*M*) is set as the ratio between majority samples and minority samples in the training data. The random forest algorithm is used to train each inspector and the threshold of uncertainty score is a tuning parameter with default value at 0.5. The resampled data passing through the inspection is further integrated with the original training data to train a final classification model. The Algorithm 1 in Fig. 3 summarizes the proposed SASMOTE method including adaptive nearest neighborhood selection and self-inspection.

## Results

### Datasets and experimental setup

The proposed method is applied to two classification problems in healthcare, where one dataset was curated for risk gene discovery of autism spectrum disorder (ASD) (Case 1) [5] and the other dataset was collected for fatal congenital heart disease (CHD) prediction (Case 2) [25].

*Case 1:* ASD is a complex neurodevelopmental condition with a strong genetic basis, but the set of disease-associated genes implicated so far is still far from complete. To enhance the discovery of ASD genes, this study develops a classification-based approach to predict ASD risk genes using features from spatiotemporal gene expression patterns in human brain, gene-level constraint metrics, and other gene variation features [5]. The dataset was acquired from the BrainSpan atlas which is a foundational resource for studying transcriptional mechanisms involved in human brain development [5]. The dataset includes 1084 genes with 121 (11%) of them are ASD associated and 963 (89%) of them are irrelevant genes. Each gene contains 553 features representing the gene’s spatiotemporal expression value in 13 developmental stages in 31 brain regions, topological features from gene coexpression networks, and gene-level constraint metrics that quantify the sensitivity of genes to variations.

*Case 2:* This study establishes a classification model to predict CHD risk using features from patient characteristics and lifestyle [25]. The dataset was obtained from the Atherosclerosis Risk in Communities (ARIC) study which contains the Cohort Component and a Community Surveillance Component for four communities: (1) Forsyth Country, NC; (2) Jackson, MS; (3) Suburban Minneapolis, MN; and (4) Washington Country, MD [26]. The dataset size in this study is 10774 with 677 (6%) of them are patients diagnosed with CHD and 10097 (94%) of them are normal patients. Each patient data contains 37 features representing patient characteristics and lifestyle behavior. Patient characteristics describe a patient using educational level, sex, age, heart rate, blood pressure, etc. The lifestyle behavior records the daily activities of the patients such as alcohol intake, smoking status, total activity hours per week, etc.

To deal with the imbalanced datasets in two classification problems, the proposed SASMOTE algorithm is applied to oversample minority class such that the imbalanced ratio between the minority and majority classes is reduced to 50%. To demonstrate the advantages of SASMOTE, the proposed algorithm is compared with other four SMOTE-based resampling algorithms, SASMOTE without visible neighbor selection (SASMOTE w/o visible), SASMOTE without inspection (SASMOTE w/o inspection), SMOTE and B-SMOTE. The classification model built on original imbalanced dataset is also considered as the baseline. In total, six classifiers are trained on the two datasets and compared in terms of prediction accuracy.

### Evaluation metrics

In the experiment, the resampling algorithms are applied to the training data and the Random Forest algorithm is used to learn the classification models on each resampled training dataset. The Random Forest algorithm has shown its advantage over the other classification models on both risk gene discovery [5] and CHD prediction [27]. The prediction accuracy of each classification model is evaluated through precision, recall and F-1 score. The 5-fold cross validation is applied to obtain the average and variation of model performance over different replications.

### Prediction performance

Tables 1 and 2 compare the prediction accuracy of six different models on risk gene discovery and CHD risk prediction respectively. Compared with the classification accuracy on original imbalanced data, all SMOTE-based resampling algorithms enable to enhance the precision and F1 score of the risk gene discovery and CHD risk prediction. This demonstrates the importance and benefits of oversampling the minority class in imbalanced data classification. Compared with the other SMOTE algorithms, three proposed algorithms further enhance the performance of precision, recall and F1 score. For the risk gene discovery case, based on the results in Table 1, the SASMOTE method is on average 7% better than B-SMOTE and 11% better than SMOTE for the F1 score. Also, it is on average 6% better than B-SMOTE and 10% better than SMOTE for the recall score. For the CHD risk prediction case, based on the results of Table 2, the SASMOTE method is on average 3% better than B-SMOTE and 6% better than SMOTE for the F1 score. It is on average 9% better than B-SMOTE and 16% better than SMOTE for the recall score as well. This demonstrates the importance and effectiveness of incorporating the visible neighbor selection and inspection. Due to the heterogeneous distribution of data in different folds of cross validation and the randomness in resampling, the model performance has high standard deviation in both tables. The standard deviation in the performance of SASMOTE without inspection is higher than the other two proposed models, which indicates that the uncertainty via self-inspection algorithm is critical for reducing the randomness in resampling and enhance the robustness in classification.

**Table 1:** For case 1: Average F1 scores, precisions, and recalls of the risk gene prediction models built on SASMOTES, SASMOTE without visible neighbors, SASMOTE without inspections, B-SMOTE, SMOTE, and original datasets. The values in brackets represent the standard deviation (Std) in 5-fold cross validation. The proposed SASMOTE performs better on the average recall and F1 score, and the original dataset performs best on the average precision

Model	Precision % (Std)	Recall % (Std)	F1 score % (Std)
SASMOTE	52.57 (8.19)	50.05 (5.38)	51.16 (6.32)
SASMOTE w/o invisible	50.34 (10.10)	49.37 (10.06)	49.72 (9.67)
SASMOTE w/o inspection	53.02 (12.30)	47.92 (11.80)	50.26 (11.94)
B-SMOTE	49.12 (10.16)	47.20 (8.76)	47.80 (8.12)
SMOTE	46.85 (11.89)	45.47 (10.97)	46.07 (11.19)
Original data	68.53 (9.31)	22.64 (6.06)	33.83 (7.70)

**Table 2:** For case 2: Average F1 scores, precisions, and recalls of the CHD risk prediction models built on SASMOTES, SASMOTE without visible neighbors, SASMOTE without inspections, B-SMOTE, SMOTE, and original datasets. The values in brackets represent the standard deviation (Std) in 5-fold cross validation. The proposed SASMOTE performs better on the average F1 score, and the SMOTE performs best on the average precision. The original dataset performs best on the average recall

Model	Precision % (Std)	Recall % (Std)	F1 score % (Std)
SASMOTE	26.06 (4.12)	25.06 (3.31)	25.41 (2.86)
SASMOTE w/o visible	26.54 (3.16)	24.45 (2.51)	25.33 (1.85)
SASMOTE w/o inspection	26.04 (3.82)	23.32 (4.01)	24.47 (3.20)
B-SMOTE	27.32 (3.70)	22.94 (3.97)	24.72 (2.64)
SMOTE	27.34 (2.11)	21.58 (3.74)	23.94 (2.24)
Original data	6.28 (4.73)	1 (0)	11.82 (0.84)

**Fig. 4:**
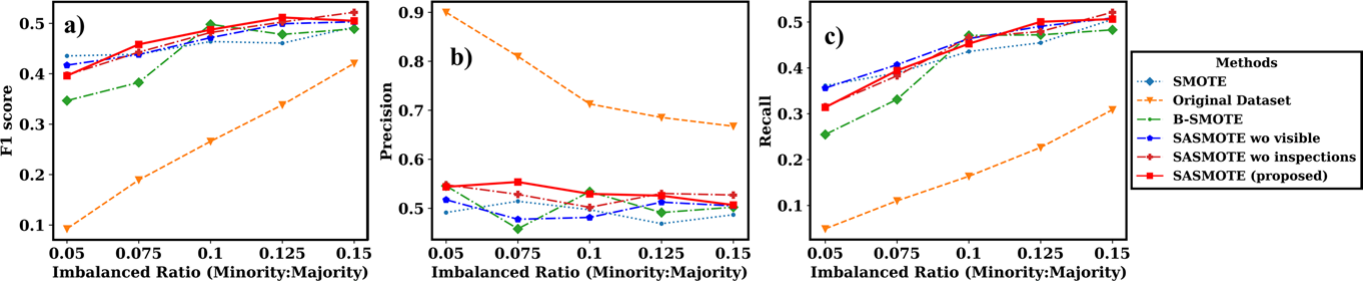
For case 1: **A** F1 scores, **B** precisions and **C** recalls of the risk gene prediction models built on SASMOTE, SASMOTE without invisible neighbors (SASMOTE w/o visible), SASMOTE without inspections (SASMOTE w/o inspections), B-SMOTE, SMOTE, and without data resampling under different balanced ratios. The proposed SASMOTE performs better on the average F1 score under most imbalanced ratios. The original dataset performs best on the average precision, but worst on the recall and F1 score

**Fig. 5:**
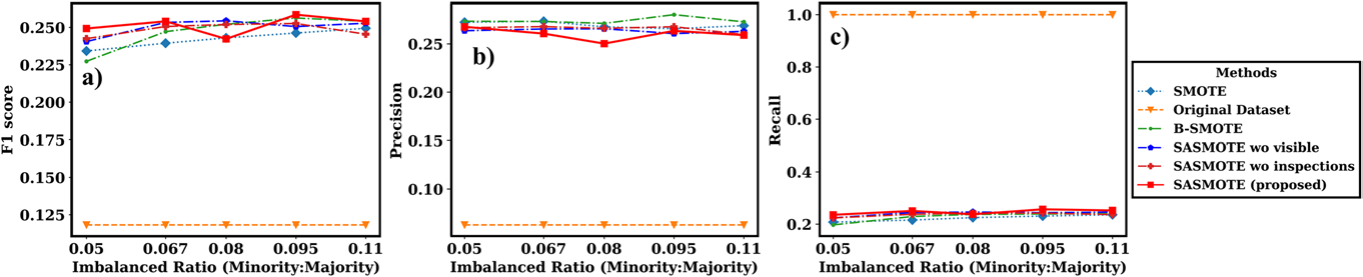
For case 2: **A** F1 scores, **B** precisions and **C** recalls of the CHD risk prediction models built on SASMOTE, SASMOTE without invisible neighbors (SASMOTE w/o visible), SASMOTE without inspections (SASMOTE w/o inspections), B-SMOTE, SMOTE, and without data resampling under different balanced ratios. The proposed SASMOTE performs better on the average F1 score under most imbalanced ratios. The B-SMOTE performs best on the average precision. The original dataset performs best on the average recall, but worst on the precision and F1 score

To evaluate the performance of the proposed method under different imbalance ratio, we further simulate the training datasets with imbalanced ratio (number of minority samples/number of majority samples) ranging from 5% to 15% from the original data for Case 1 and 5% to 11% for Case 2. The datasets with imbalanced ratio lower than the original data are simulated by down sampling the minority data while the datasets with imbalanced ratio higher than the original data are simulated by down sampling the majority data. The performance of the SASMOTE algorithm and other benchmark models is illustrated in Figs. 4 and 5. The models trained on resampled datasets have better performance than the model learnt from imbalanced data under all imbalance ratios. For the risk gene discovery, the difference is enlarged when data is highly imbalanced with 5% imbalanced ratio. For the CHD risk prediction, the difference is enlarged when data is highly imbalanced with 8% imbalanced ratio. Moreover, the proposed self-inspected adaptive SMOTE (SASMOTE) algorithm on average has better performance than other methods on F1 score under most imbalanced ratios in Figs. 4(a) and 5(a). Also, the SASMOTE without visible neighbors selection and SASMOTE without inspections on average have better F1 score than the other SMOTE algorithms under most imbalanced ratios, indicating the effectiveness of the adaptive nearest neighborhood selection algorithm and the inspection. Figure 4(b) shows that SAMOTE has better precision score on average than the other resampling methods under most imbalanced ratios. Figure 5(c) further shows the effectiveness of the proposed SASMOTE method since it leads to higher recall compared to the other resampling algorithms on average.

### Sensitivity Analysis

To explore how the change of parameters affects the algorithm performance, we conduct sensitivity analysis on three different parameters including the number of nearest neighbors *K* (% of the number of minority samples), the number of samples generated from each minority data *N*, and the threshold of uncertainty score *T* (%). The results shown in Tables 3, 4, and 5 are based on the experiments on the risk gene discovery study.

**Table 3:** Average F1 scores on different *K* values. The values in brackets represent the standard deviation (Std) in 5-fold cross validation. The proposed SASMOTE algorithms perform better on the average F1 score with respect to the value of K ranging from 10% to 75% while SMOTE performs better with respect to the value of *K* = 5%

Model	$\boldsymbol{K = 5\%}$ (Std)	$\boldsymbol{K = 10\%}$ (Std)	$\boldsymbol{K = 25\%}$ (Std)	$\boldsymbol{K = 50\%}$ (Std)	$\boldsymbol{K = 75\%}$ (Std)
SASMOTE	49.15 (9.02)	47.57 (9.80)	51.38 (8.49)	51.30 (5.99)	51.16 (6.32)
SASMOTE w/o visible	49.71 (9.79)	49.69 (9.82)	49.95 (7.58)	48.11 (7.76)	49.72 (9.67)
SASMOTE w/o inspection	46.81 (9.63)	48.57 (6.97)	50.38 (8.64)	51.38 (12.29)	50.26 (11.94)
B-SMOTE	48.84 (9.20)	48.63 (7.61)	48.02 (8.50)	46.14 (7.43)	47.80 (8.12)
SMOTE	49.73 (10.09)	47.84 (9.30)	50.15 (10.49)	48.69 (6.58)	46.07 (11.19)

**Table 4:** Average F1 scores on different *N* values. The values in brackets represent the standard deviation (Std) in 5-fold cross validation. The proposed SASMOTE algorithms perform better on the average F1 score with respect to the value of *N* = 1, 2, 3, 5 while B-SMOTE performs better with respect to the value of *N* = 4

Model	$\boldsymbol{N = 1}$ (Std)	$\boldsymbol{N = 2}$ (Std)	$\boldsymbol{N = 3}$ (Std)	$\boldsymbol{N = 4}$ (Std)	$\boldsymbol{N = 5}$ (Std)
SASMOTE	47.04 (7.22)	51.32 (7.70)	51.88 (6.60)	51.13 (8.51)	51.16 (6.32)
SASMOTE w/o visible	48.97 (7.90)	49.60 (10.14)	48.04 (8.48)	50.15 (8.55)	49.72 (9.67)
SASMOTE w/o inspection	48.85 (4.60)	48.30 (6.90)	53.34 (6.92)	46.56 (8.90)	50.26 (11.94)
B-SMOTE	44.76 (7.85)	49.37 (10.60)	49.31 (7.76)	51.17 (10.53)	47.80 (8.12)
SMOTE	45.59 (8.90)	46.31 (9.71)	47.72 (11.81)	50.03 (7.92)	46.07 (11.19)

**Table 5:** Average F1 scores on different threshold of uncertainty score *T* values. The values in brackets represent the standard deviation (Std) in 5-fold cross validation. The proposed SASMOTE algorithm performs better on the average F1 score with respect to the value of T ranging from 25% to 60% while the SASMOTE without visible neighbors performs better with respect to the value of *T* = 75% and *T* = 90%

Model	$\boldsymbol{T = 25\%}$ (Std)	$\boldsymbol{T = 50\%}$ (Std)	$\boldsymbol{T = 60\%}$ (Std)	$\boldsymbol{T = 75\%}$ (Std)	$\boldsymbol{T = 90\%}$ (Std)
SASMOTE	50.45 (9.12)	51.16 (6.32)	49.63 (7.22)	48.44 (7.04)	47.06 (6.65)
SASMOTE w/o visible	49.26 (8.76)	50.04 (8.22)	47.39 (7.48)	49.72 (9.67)	49.51 (7.31)

Table 3 illustrates the average F1 scores on different *K* values. It shows that when *K* is extremely low (i.e., 5%), the F1 scores on all SMOTE algorithms do not differ significantly. However, this is not commonly used in reality as the number of nearest neighbors should be larger than the number of samples to be generated [9]. For the other *K* values, the proposed SASMOTE algorithms always have higher average F1 scores than the other SMOTE-based algorithms, which indicates the robustness of the proposed algorithm with respect to the value of *K*. When *K* is relatively large (i.e., 50% and 75%), the SASMOTE algorithms with inspection have much lower standard deviations than the other resampling algorithms, which indicates the importance and effectiveness of quantifying the uncertainty of resampled data via inspection when *K* is large.

Table 4 represents the average F1 scores on different *N* values. It showcases that SASMOTE methods on average has better performance than other SMOTE-based algorithms under different settings of *N*. Even though they do not achieve the best F1 score in the case of $N = 4$, they still have lower standard deviations than the B-SMOTE algorithm. This indicates the effectiveness and robustness of the SASMOTE algorithms regardless of the size of synthetic minority samples. It also demonstrates the power of sample filtration from inspectors when the number of synthetic minority samples increases.

Table 5 compares the performance of SASMOTE method and SASMOTE without visible neighbors under different threshold of uncertainty score (*T*). SASMOTE performs better than SASMOTE without visible neighbors on average when the threshold is between 50% to 60% while it performs worse when the threshold is between 75% to 90%. This experiment suggests that SASMOTE without visible neighbors needs higher threshold of uncertainty since the low-quality samples can be generated from the invisible nearest neighbors. SASMOTE, on the other hand, needs lower threshold of uncertainty because the samples generated from the visible neighbors have higher quality. The average performance of both methods increases with respect to the threshold of uncertainty score first and then decreases. This is due to the high threshold of uncertainty score can lead to smaller number of training samples, which potentially influences the classification accuracy. Therefore, 50% is the optimal threshold of uncertainty score for both SASMOTE and SASMOTE without visible neighbors algorithms.

## Discussion

The resampling techniques, especially SMOTEs, are popular for solving imbalanced data classification problem in healthcare. However, existing SMOTE algorithms may generate “uncertain” samples that fall into to the wrong class due to the lack of selection of nearest neighbors used for sample generation and the lack of inspection of generated samples. To close this gap, this paper proposes a self-inspected adaptive SMOTE (SASMOTE) algorithm. The proposed algorithm introduces an adaptive nearest neighborhood selection algorithm to identify the “visible” neighbors for generating more accurate minority class samples. An uncertainty via inspection approach is further developed in the proposed method to measure the quality of resampled data and filter out the low-quality ones. The proposed method is capable of generating high-quality resampled data, further improving the performance of machine learning models on highly imbalanced healthcare data and advancing the development of biomedical data mining.

By applying the proposed SASMOTE method to two real-world healthcare datasets, risk gene discovery and fatal congenital heart disease prediction, the SASMOTE method on average has better performance than the other SMOTE-based resampling algorithms, which demonstrates the advantages of using the “visible” neighbors and uncertainty via inspection. By comparing the SASMOTE method with and without self-inspection algorithm, the uncertainty via self-inspection algorithm is found to be critical for reducing the variance in resampling and improving the robustness in classification. Furthermore, the SASMOTE method shows advantage on the average performance over other SMOTE-based resampling algorithms under different imbalanced ratios, and the advantage is more significant when the dataset is highly imbalanced. Through the sensitivity analysis, we explored the effects of hyperparameters, including the number of nearest neighbors, number of generated samples, and threshold of uncertainty score, on the SASMOTE performance. The SASMOTE method is preferred when the number of nearest neighbors is relatively large and the threshold of uncertainty score needs to be carefully tuned for achieving better average performance.

The two case studies conducted in this study may not be able to cover all types of imbalanced data classification problems in healthcare. In the future, we will apply the proposed SASMOTE method to other applications, such as the rare disease prediction [28, 29]. The proposed method is evaluated on two healthcare datasets without missing values. However, the missing value issue is commonly observed in most real healthcare datasets [30, 31]. Thus, we will also explore the robustness of the proposed method when facing with the missing values.

## Data Availability

The data and code that support the findings of this study are available on https://shines-lab.github.io//projects/.
